# University of Louisville

**Published:** 1851-10

**Authors:** 


### THE WESTERN JOURNAL.

THIRD SERIES.—VOL. VIII.—NO. IV.

LOUISVILLE, OCTOBER 1, 1851.

### UNIVERSITY OF LOUISVILLE.

Professor Silliman, who returned from Europe in the steam ship Pacific, on the 15th of September, made some valuable purchases for the University of Louisville while abroad, consisting of books, chemical apparatus, and models illustrative of healthy and morbid anatomy. The articles are expected to arrive in time for the approaching course of lectures. Prof. S. mentions that the University of Pennsylvania, and the schools of New Orleans, St. Louis, Columbus, and Nashville had made purchases of similar models, which are prepared by Madame Thibert, widow of the late Dr. Thibert, an American lady. They are said to be of exquisite workmanship, rendering and preserving the minutest anatomical details. "Besides the excellence of these models for instruction," says Dr. S.—"all copied from nature and vastly superior in effect and usefulness to the nauseous and ill-conditioned wet specimens which usually disgrace museums, they form a collection which it costs nothing to keep." The series of models from which Dr. S. made his selections embraces all the pathological specimens in the museum of Dupuytren. The addition of these fine models to our schools will very much enhance the pleasure and interest both of medical teaching and study.

